# Gram-positive bacterial membrane lipids at the host–pathogen interface

**DOI:** 10.1371/journal.ppat.1011026

**Published:** 2023-01-05

**Authors:** Luke R. Joyce, Kelly S. Doran

**Affiliations:** University of Colorado Anschutz Medical Campus, Department of Immunology and Microbiology, Aurora, Colorado, United States of America; Tufts Univ School of Medicine, UNITED STATES

## Introduction to gram-positive membrane lipids

**Fig 1:**
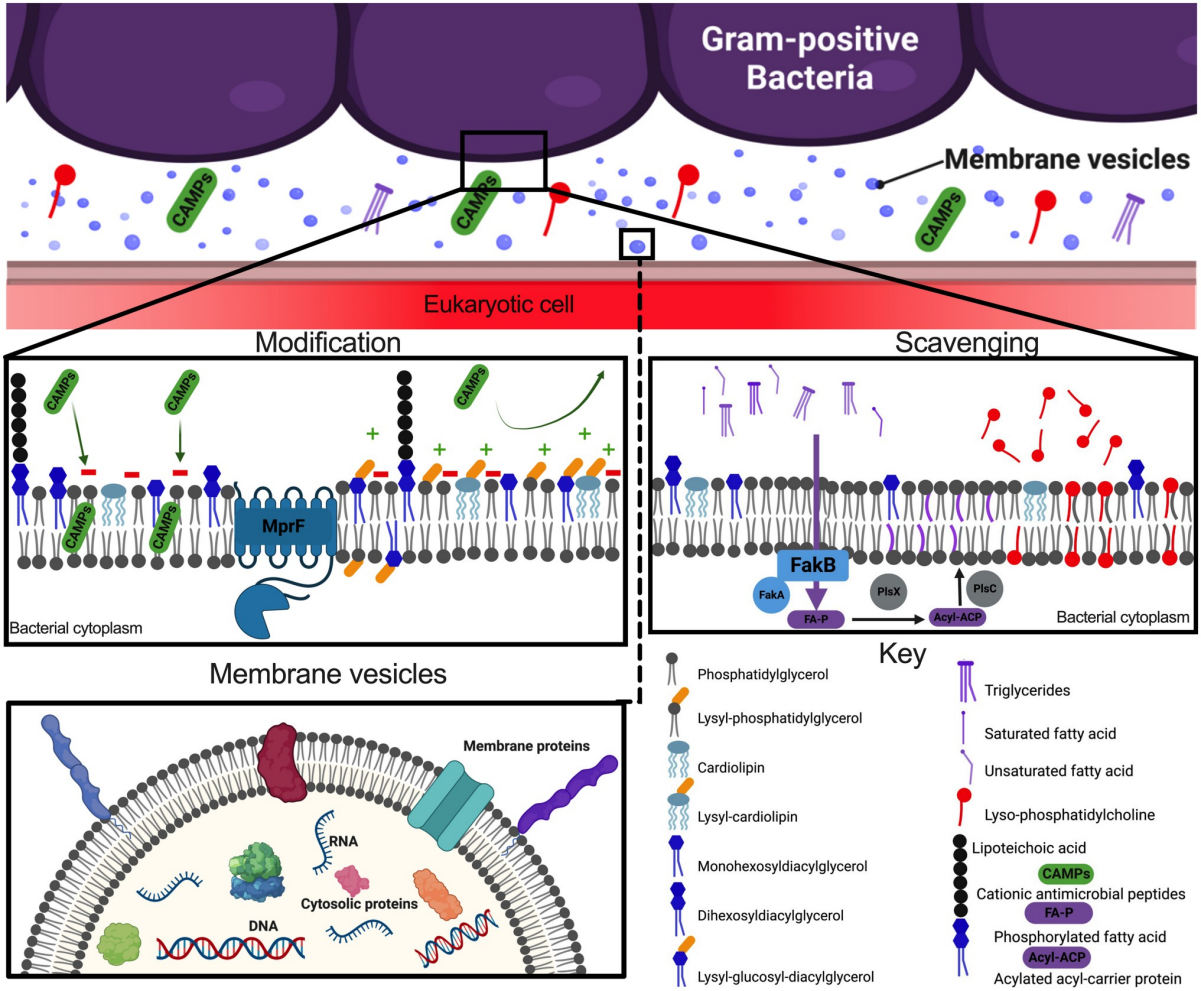
Gram-positive membrane lipids at the host–pathogen interface. Gram-positive bacteria utilize the cellular membrane during host–pathogen interactions. Modification of lipids protects against antimicrobials, scavenging host lipids and fatty acids provides protection against membrane stress and provides a form of membrane mimicry, and MVs carry a variety of cargo from the bacterial cell into the surrounding environment to interact with host cells. MprF; multiple peptide resistance factor, FakB; fatty acid kinase B, FakA; fatty acid kinase A, PlsX; phosphate acyltransferase, PlsC; 1-acyl-sn-glycerol-3-phosphate acyltransferase. Figure made by BioRender.com.

A critical site of interaction between bacterial pathogens and the host is the bacterial cellular membrane. In gram-positive bacteria, the cellular membrane is beneath a thick peptidoglycan cell wall and potentially a capsule depending on the species. The cellular membrane is comprised of a bilayer of lipids that are vital for membrane protein folding and localization, cellular replication, virulence factors, and stress survival [1]. Modulation of the bacterial membrane can provide beneficial effects and aid survival depending on the microenvironment surrounding the bacteria. The 2 major lipid classes with distinct roles and properties are phospholipids and glycolipids, which are vital to bacterial survival. Both lipid classes possess the hydrophobic diacylglycerol (DAG) base, comprised of acyl chains or fatty acids connected via a glycerol backbone. Phospholipids have headgroups attached to DAG via a phosphate group. Whereas glycolipids have hexoses, such as glucose or galactose, as headgroups connected directly to DAG. More is currently known about phospholipids and the physiological role they exert such as membrane fluidity and extracellular charge, while less is known about the function of glycolipids, other than to act as lipid anchors for the major gram-positive polymer, lipoteichoic acid (LTA) [1]. Membrane modifications have been identified in a variety of gram-positive bacteria [1], and the major lipids detected in the membrane of these bacteria are phosphatidylglycerol (PG), cardiolipin (CL), monohexosyldiacylglycerol (MHDAG), dihexosyldiacylglycerol (DHDAG), and amino-acylated lipids [2–6] (Table 1). This review will primarily focus on the well-studied, extracellular, and non-sporulating pathogenic species in the genera of Staphylococci, Enterococci, and Streptococci. Here, we will highlight the major advances in our understanding of lipid function and host interactions, including amino-acylation modifications, membrane mimicry via host metabolite scavenging, and membrane vesicles (MVs) (Fig 1).

**Table 1:** Characterized lipids of select gram-positive bacteria.

Species	Lipids
*Staphylococcus aureus*	PG, CL, Lys-PG, MHDAG, DHDAG
*Enterococcus faecalis*	PG, CL, Lys-PG, MHDAG, DHDAG
*Enterococcus faecium*	PG, CL, Lys-PG, MHDAG, DHDAG
*Streptococcus pneumoniae*	PG, CL, PC, MHDAG, DHDAG
*Streptococcus pyogenes*	PG, CL, PC*, MHDAG, DHDAG
*Streptococcus agalactiae*	PG, CL, pPC*, Lys-PG, MHDAG, DHDAG, Lys-Glc-DAG
*Listeria monocytogenes*	PG, CL, Lys-PG, Lys-CL, PI, MHDAG, DHDAG
*Clostridioides perfringens*	PG, PE, Aln-PG, Lys-PG, PL, MHDAG, DHDAG
*Bacillus subtilis*	PG, CL, PE, Lys-PG, MHDAG, DHDAG, GPL

See references [1,4,5,10] for lipid identifications. PG, phosphatidylglycerol; CL, cardiolipin; Lys-PG, Lysyl-PG-PG; Aln-PG, Alynyl-PG; Lys-CL, Lysyl-CL; PC, phosphatidylcholine; pPC, plasmanyl-PC; PE, phosphatidylethanolamine; PI, phosphatidylinositol; PL, plasmalogens; MHDAG, monohexosyldiacylglycerol; DHDAG, dihexosyldiacylglycerol; Lys-Glc-DAG, lysyl-glucosyl-diacylglycerol; GPL, glycophospholipid.

“*” denotes lipids detected when cultured in the presence of human serum [5].

## Amino-acylation modifications: One size doesn’t fit all

The host environment can be perilous for bacteria as the microenvironment is continually changing. Bacteria will encounter fluctuations in pH, temperature, osmotic changes, as well as the immune system and antimicrobial agents designed to protect the host. To overcome these hostilities, bacteria can modify lipids to alter their physical properties. A well-known example is the amino-acylation of PG with the positively charged amino acid lysine to form Lysyl-PG [7]. The amino acids alanine or arginine may also be used but is less prevalent in gram-positive bacteria and is species dependent [1]. The enzyme responsible for amino-acylating lipids is the multiple peptide resistance factor (MprF), first identified in *Staphylococcus aureus* [8], is present in Staphylococcal, Streptococcal, and Enterococcal genera [7]. MprF is a bifunctional enzyme containing an N-terminus flippase domain and a C-terminus catalytic domain that obtains an amino acid from a charged tRNA and facilitates the amino-acylation of the lipid headgroup [9]. MprF is constitutively active; however, environmental conditions have been shown to induce increased activity to aid survival [10]. Aminoacylation is not specific to PG and in the case of *Listeria*, Lys-CL is observed [10]. Furthermore, the amino-acylated glycolipid, lysyl-glucosyl-diacylglycerol (Lys-Glc-DAG) was recently identified in *Streptococcus agalactiae* (Table 1) [11]. In general, amino-acylation aids in masking the negative charge associated with the extracellular surface of the membrane that is attributed to the phosphate headgroup of phospholipids, which has beneficial implications for human pathogens.

Altering the surface charge of the cellular membrane results in repulsion of cationic antimicrobial peptides (CAMPs) produced by the host, such as LL-37, or antibiotic’s such as Daptomycin that target the bacterial membrane [8,12] (Fig 1). To study amino-acylated lipid contributions, Δ*mprF* mutants have been constructed, but have varied phenotypes in different pathogens. *S*. *aureus* Δ*mprF* strains are significantly attenuated in blood survival in vivo, whereas Enterococcal and Streptococcal strains were not [8,11,13]. Removal of Lys-PG from the membrane of Staphylococci and Enterococci resulted in a significant reduction of the negative extracellular surface charge creating a more neutral surface charge, which decreased susceptibility to CAMPs, antibiotics, and opsonophagocytic killing by neutrophils [8,13,14]. Removal of both Lys-PG and Lys-Glc-DAG from the membrane of *S*. *agalactiae* led to reduced growth in low pH conditions and significantly less invasion of human brain endothelial cells in vitro but no change in susceptibility to CAMPs and other antimicrobials [11,15]. *S*. *agalactiae* Δ*mprF* strains exhibited similar bloodstream survival as WT strains; however, the pathogenesis of meningitis and blood–brain barrier (BBB) crossing was significantly attenuated suggesting the lysine lipids are important for interaction with the BBB [11]. While Staphylococci may use Lys-PG to resist neutrophil killing during bloodstream infections, Enterococci and Streptococci may utilize LTA as the major factor to survive neutrophil defenses [15]. These differences identify the importance of investigating modified lipids in context of an in vivo infection and mechanistic differences between bacterial species.

## Cellular membrane mimicry: A wolf in sheep’s clothing?

An interesting area of research is the scavenging and utilization of host-derived metabolites such as fatty acids and full lipid species, such as phospholipids, from sera, other biological fluids, and the native microbiome [16]. Enterococci and Staphylococci only scavenge host fatty acids and not full lipid species to incorporate into their cellular membrane, potentially impacting membrane fluidity, which aid survival during temperature shifts, pH changes, and antibiotic conditions associated with infection [16–18]. Scavenging of fatty acids have been demonstrated in Staphylococci, Enterococci, and Streptococci through use of the fatty acid kinase (Fak) machinery [16,18–20] (Fig 1). Interestingly, when *S*. *aureus* is cultured in the presence of human serum, an abundant level of host fatty acids and full lipid species have been found to associate with the extracellular surface, but not be incorporated into the cellular membrane [18]. The mechanistic nature of how the host lipids associate to the extracellular surface is currently unknown.

Streptococci scavenge both fatty acids and host lipids [5,19]. *S*. *pneumoniae*, *S*. *pyogenes*, and *S*. *agalactiae* scavenge lyso-phosphatidylcholine (Lyso-PC; a PC lipid headgroup with a single acyl chain abundant in human sera and a signaling molecule [21]) and add the second acyl chain to it to form PC [5]. Additionally, *S*. *pneumoniae* and other mitis group streptococci scavenge glycerophosphocholine, another major human metabolite, and acylate it to from PC [22]. The incorporation of PC, a zwitteronic phospholipid, into the Streptococcal membrane potentially alters the extracellular charge and may increase the ability to survive host stresses like amino-acylated lipids. PC is a major component of human cellular membranes [21]. Thus, the ability to utilize host metabolites, such as host fatty acids and lipid species, on the bacterial cell surface may represent a form of rudimentary cellular mimicry to protect against the immune response to pathogenic bacteria and other physiological stresses [23]. The full implications of gram-positive bacteria incorporating host-derived fatty acids, lipid classes, and association of host lipids on the cellular surface of pathogens remain to be further investigated.

## Membrane vesicles: Intentional or random event?

An exciting area of novel research in the gram-positive bacterial field is the formation and function of MVs, also known as extracellular vesicles. MVs are membrane derived, nanosized, bilayer spheres that are packaged with a variety of cargo such as proteins, nucleic acids, and additional compounds, that are then released into the surrounding environment [24,25] (Fig 1). MVs were previously thought to not be produced by gram-positive bacteria due to the thick peptidoglycan cell wall or if a capsule is present. The mechanistic biogenesis of MVs is unknown. Is it an intentional cellular program to direct cargo to the extracellular environment or a random event due to biophysical properties of lipid membranes? Multiple factors such as membrane fluidity, proteins, and large genetic networks are shown to influence MV production [24]. Even though MV production is ubiquitous; factors like membrane stress by antimicrobial peptides, growth phase changes, and temperature have been shown to alter the amount of MVs produced and the type of cargo packaged, thus improving the bacteria’s ability to survive in harsh conditions and aiding disease progression [24,26–33].

MVs are potent activators of the host immune system, inducing cytokine release and immune cell activation [30–33]. *S*. *pneumoniae* MVs activate macrophages (M2) that causes increased bacterial phagocytosis; interestingly, MV-activated macrophages allowed for better survival of *S*. *pneumoniae* within macrophages [30]. *S*. *agalactiae* MVs protect against reactive oxygen species in vitro and induce adverse pregnancy results in vivo due to inflammation in the uterus [29,31]. Staphylococcal MVs are known to activate inflammation via TLR2-dependent mechanisms due to the high abundance of lipoproteins present in the MVs [32], and both Streptococcal and Enterococcal MVs modulate the NF-KB pathways in macrophages [30,34]. Immune activation by MV’s could be beneficial, as is the case for *S*. *pneumonaie* described above, detrimental to the bacteria, or a combination of both, although these mechanisms are yet to be elucidated. The gram-positive MV field is still in its infancy, and the full implications of MVs on disease progression will be an exciting topic for further investigation.

## Conclusion

The gram-positive cellular membrane and its lipid component is an under explored and important site of interaction during pathogenesis. We have highlighted a variety of important roles the bacterial cellular membrane and its components play during pathogenesis. We propose the biosynthetic enzymes associated with lipids and lipid modifications may be potential targets for therapeutics, either alone or in conjunction with current treatment strategies, which has recently been demonstrated [35]. Furthermore, we posit that exploration of amino-acylated lipids is important to understand the varying mechanisms different gram-positive bacterial species utilize to survive during growth in the human host and that the same lipids in different species are utilized differently in a “no one size fits all” approach. Similarly, different strains within a given species may vary in uptake and usage, MV production and cargo, and lipid modifications based on unique genetic variations between strains. Further exploration of gram-positive membrane components and lipids are needed, with exciting possibilities to identify and characterize unknown lipids and pathways important for pathogenesis.
